# Nazheng stretch-rotation manipulation in the treatment of atlantoaxial subluxation in children: A case report

**DOI:** 10.1097/MD.0000000000042178

**Published:** 2025-05-30

**Authors:** Jiahe Liu, Hongpeng Lin, Qingjun Zhang, Yi Zhou, Jiayi Liu, Chenxi Qi, Wei Qi

**Affiliations:** a Department of Acupuncture and Tuina, Changchun University of Chinese Medicine, Changchun, China; b Department of Orthopedics, Hubei University of Chinese Medicine, Hubei, China; c Tuina Department, Shenzhen Hospital of Integrated Traditional Chinese and Western Medicine, Shenzhen, China; d Department of Traditional Chinese Medicine, Liaoning University of Traditional Chinese Medicine, Shenyang, China; e Traditional Chinese Medicine Orthopedics and Traumatology Department, Shenzhen Baoan Authentic TCM Therapy Hospital, Shenzhen, China.

**Keywords:** atlantoaxial subluxation, case report, manual manipulation, Nazheng stretch-rotation manipulation

## Abstract

**Rationale:**

Atlantoaxial subluxation in children refers to a kind of disease characterized by neck deviation, pain, and limited activity as the main clinical manifestations resulting from dysfunction of the atlantoaxial joint due to trauma, poor posture, inflammation, or other etiological factors. In this paper, according to the special anatomical structure of the atlantoaxial joint, combined with the distinct physiological and pathological characteristics of pediatric development, the diagnostic criteria of atlantoaxial subluxation in children was supplemented, and a safer, painless, and easy-to-operate Nazheng stretch-rotation manipulation was proposed and applied to treat the disease, in order to provide new ideas and new methods for clinical diagnosis and treatment of atlantoaxial subluxation in children.

**Patient concerns:**

An 8-year-old Chinese girl was the patient. The patient presented with neck pain, cervical deviation, and restricted range of motion for over 2 months, accompanied by left flexion to right rotation, and the flexion and extension of the cervical spine, as well as the left and right rotation were clearly limited.

**Diagnoses:**

Atlantoaxial subluxation.

**Interventions:**

Given the patient’s condition, we used the Nazheng stretch-rotation manipulation as the primary therapeutic intervention, supplemented by traditional Chinese medicine hot compress.

**Outcomes:**

After 4 days of treatment, a complete resolution of cervical pain was observed, with restoration of cervical shape, although flexion-extension and rotational movements remained mildly limited. After 11 days of treatment, the cervical range of motion had significantly improved, with near-complete restoration of flexion-extension and rotational mobility. After 20 days, both cervical morphology and functional mobility had returned to within normal physiological parameters.

**Lessons:**

Given the limited therapeutic options currently available for this disease, the clinical practice of this case confirmed that the reduction of atlantoaxial subluxation does not necessarily need to use the pulling method. As long as the periarticular soft tissues are fully released, the atlantoaxial joint can be reseted with minimal force during the stretching process. If this method can be replicated and promoted, it will further improve the safety parameters of manual treatment of atlantoaxial subluxation in children, and it will be more easily accepted by children and parents.

## 1. Introduction

Atlantoaxial subluxation in children refers to a kind of disease characterized by neck deviation, pain, and limited activity as the main clinical manifestations resulting from dysfunction of the atlantoaxial joint due to trauma, poor posture, inflammation, or other etiological factors.^[1,2]^ Analysis of the patient’s clinical symptoms, often with spontaneous torticollis, limited rotational activity, upper neck pain, and other associated manifestations.^[3]^ Modern studies have found that children’s developing bodies contributes to their joint capsule relaxed, ligaments elastic, atlanto-axial joint concave shallow; the atlanto-axial joint and facet joint are nearly coplanar, and the neck muscle group is underdeveloped, and the external stability of the atlanto-axial joint is poor. In addition, after children suffer from respiratory tract infection, the inflammatory exudate induced by infection can directly reach the atlantoaxial joint capsule and cause vasodilation, or in otolaryngology surgery, the intraoperative traction or postoperative inflammatory reaction can directly or indirectly lead to atlantoaxial joint ligament relaxation. These are also the main causes of Grisel syndrome.^[4]^ Based on the aforementioned physiological and pathological basis, coupled with the characteristics of children’s hyperactivity, minimal head movement or minor external force may lead to displacement of the odontoid process from the transverse ligament of the atlas, thereby inducing atlantoaxial joint subluxation. Subsequently, the vertebral arteries within the transverse foramina bilateral to the atlantoaxial spinal body become compressed, resulting in compromised cerebral blood supply. which can lead to symptoms such as dizziness, bloating, tinnitus, and abnormal blood pressure; concurrently, the soft tissue on both sides of the cervical spine is unbalanced, accompanied by localized muscular spasms; and the head movement is limited, and the nerves of the neck are also stimulated. Thus, a series of uncomfortable symptoms such as headache and posterior occipital pain are also accompanied.^[5]^

At present, the atlantoaxial subluxation in children represents a relatively common clinical entity in pediatric practice. Traction and manual therapy are the first clinical treatments, and surgical treatment is used for those who are ineffective. Relatively speaking, manual therapy is easier to operate, safer and more effective, without side injury and sequelae.

## 2. Case report

### 2.1. Clinical presentation

**Figure 1.**
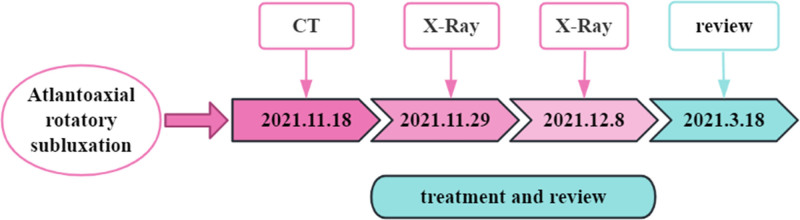
Timeline of clinical and procedural data.

The timeline of clinical and procedural data is shown in Figure 1.

**Figure 2.**
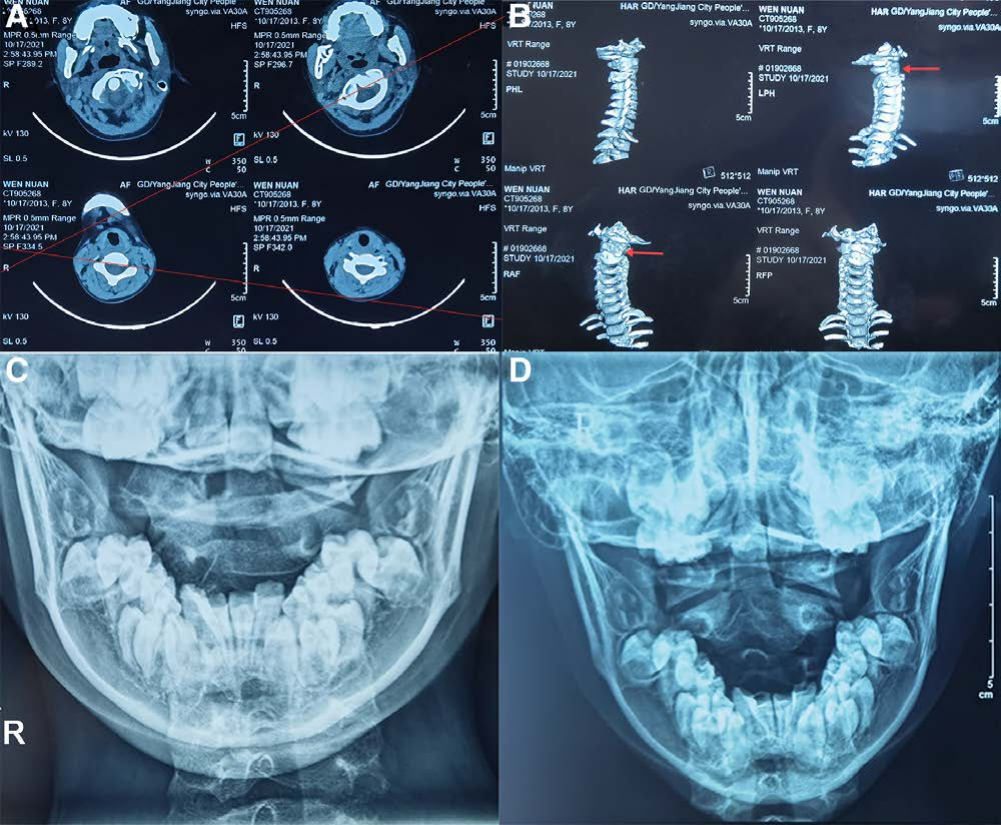
Three-dimensional CT of the cervical spine before massage treatment: (A) 3-dimensional CT of cervical spine (the angle between the 2 red lines is about 40 degrees); (B) 3-dimensional CT of cervical spine (red arrow refers to the site of rotation). X-ray of the open-mouth position cervical spine after massage treatment: (C) X-ray of the open-mouth position cervical spine (November 29, 2021 review); (D) X-ray of the open-mouth position cervical spine (December 08, 2021 review). CT = computed tomography.

An 8-year-old girl presented to our clinic with her family members. She complained of neck deviation and pain, accompanied by a rotation to the left and right; the flexion and extension of the cervical spine and the left and right rotation were significantly limited. History of past illness: the child suddenly turned her head backward from the right side when walking 2 months ago, resulting in acute neck pain and subsequent limited mobility. She had visited hospitals in Yangjiang, Guangzhou and other places, and was diagnosed as “atlantoaxial subluxation” by X-ray, magnetic resonance imaging, and 3-dimensional computed tomography (CT). Previous therapeutic interventions included cervical orthosis immobilization, topical traditional Chinese medicine application, cervical traction, among other conservative measures. While neck pain demonstrated partial relief, and cervical spine skew showed improvement, significant activity limitations persisted. Other doctors had recommended surgical treatment, but the patient did not want to do the operation and was subsequently referred to our hospital. Personal and family history: the patient denied a family history of similar diseases. Physical examination: body temperature: 36.8 °C; blood pressure: 114/70 mm Hg; heart rate: 90 bpm; respiratory rate: 18 breaths per minute. Cervical examination demonstrated torticollis with leftward curvature, rightward rotation, and severely restricted range of motion: flexion 10 degrees, extension 15 degrees, left rotation 0 degree, and right rotation 15 degrees. The transverse process of the left axis protruded posteriorly, with tenderness (+). Imaging examination: The X-ray of the open-mouth position cervical spine was nondiagnostic due to bony overlap; 3-dimensional CT (Fig. 2A and B): The atlas rotates about 40 degrees clockwise around the odontoid process of the axis. The left lateral mass is located in front of the axis vertebral body, and the right lateral mass is located behind the superior articular process of the axis. And after the rotation of the atlas, the left lateral mass fell to the front of the axis spine body, a bone overlap was produced, which was the most serious case of subluxation that we had encountered at present. Based on clinical presentation and radiographic findings, the patient was diagnosed with severe atlantoaxial subluxation (the subluxation of clockwise rotation).

### 2.2. Interventional procedure

The primary therapeutic approach is Nazheng stretch-rotation manipulation, supplemented by hot compress of traditional Chinese medicine. The adjunctive therapeutic modality of Traditional Chinese Medicine hot compress therapy aims to achieve meridian warming, cold dispersion, collateral dredging, and pain alleviation. The prescription is: dodder perilla seed, white mustard seed, psoralen and white aconite 150 g each. The basic operation process is: crushing the traditional Chinese medicine and putting it in a cotton bag or gauze bag to ensure that the bag mouth is tightened. The drug package is first heated in a steamer or microwave oven, and then waited until the temperature dropped to about 50 °C, ensuring optimal patient tolerance and safety. Then the medicine package is placed on a towel and placed on the affected area. Through the introduction of drug heat, the local blood circulation is promoted, so that the effective components of traditional Chinese medicine can penetrate the skin to the muscle striae, and the medicine package is covered with a layer of impermeable membrane to prevent the release of medicine gas. After 30 minutes of hot compress, taking off the medicine package. The treatment protocol is administered at 48-hour intervals. And no external neck support was provided after the first treatment.

By November 22nd, complete resolution of neck pain was achieved, with restoration of normal cervical shape and mild residual discomfort during cervical flexion-extension and rotation movements. Subsequent evaluation on November 29th demonstrated near-complete restoration of cervical mobility in both flexion and rotation. The X-ray of the open-mouth position cervical spine (Fig. 2C) was reviewed: The left side of the lateral atlantoaxial joint was basically aligned, and the right side was still overlapped. On December 8th, the neck shape and range of motion were as usual. The X-ray of the open-mouth position cervical spine (Fig. 2D) showed that the position of the atlantoaxial joint was basically normal, and she was recommended to be discharged. The family members were worried about instability and asked for consolidation treatment. A total of 22 treatments were performed, she was cured and discharged on the 18th. The specific operation was as follows: After the patient was in the supine position, the suboccipital muscles (mainly the rectus capitis posterior major muscle and the obliquus capitis inferior muscle) were fully released, the middle finger of the surgeon’s left hand hooked the right side of the axis spinous process and fixed it to the left, the right palm root was close to the occipital bone and stretched the cervical spine upward, and let the patient actively rotate the cervical spine to the maximum angle in the counterclockwise direction for about 5 seconds and then return to the neutral position, and then rotate again to the maximum angle for about 5 seconds, and return to the neutral position. This maneuver was repeated for 3 to 5 cycles, so as to achieve the purpose of slow reduction of the atlantoaxial joint, and then the therapeutic intervention was completed.

### 2.3. Outcome and follow-up

On March 18th, 2022, the patient was contacted by telephone 3 months after the end of treatment. No subsequent therapeutic interventions were administered during the follow-up period. The therapeutic efficacy of Nazheng stretch manipulation in conjunction with traditional Chinese medicine hot compress was still significant, with no clinical problems occurred.

## 3. Discussion

Clinically, the atlantoaxial subluxation in children is not rare. The etiologies of the disease are complex and the symptoms are numerous.^[6,7]^ It has become a serious social health problem, which has a bad impact on the health and quality of children’s lives. At present, the diagnostic criteria for the disease are not uniform, and the rate of missed diagnosis and misdiagnosis is still high. Among them, the rehabilitation department of Deyang People’s Hospital of Sichuan Province^[8]^ reported 2 cases of neck pain and limited activity without obvious inducement in a report. Before the correct diagnosis of spontaneous atlantoaxial subluxation was established, the children was initially misdiagnosed as stiff neck, and the treatment of atlantoaxial subluxation with stiff neck could lead to aggravation of the disease. In addition, in the clinical data of 146 cases of atlantoaxial subluxation,^[9]^ 84 cases were typical, and 31 cases of atlantoaxial subluxation with throat-clearing cough were misdiagnosed as allergic cough and other medical diseases. There were 18 males and 13 females, the mean age was 5.8 years (range: from 4–10 years old). All patients showed frequent throat clearing, some accompanied by slight sigh or mouth twitching. Physical examination of C1 to C2 transverse process, vertebral arch, posterior joint was 1 side uplift and tenderness, with contralateral depression and no tenderness, axis spinous process deviation, normal or congestion of the pharynx, the muscle strength and nerve reflex examination did not lead to positive signs. Cervical spine X-ray of the open-mouth position or CT showed that ADI (atlanto-dental distance) ≥ 5 mm, atlanto-dental lateral space ≥ 2 mm. The ADI exhibited characteristic “V” or inverted “V” morphological alterations. Among the 31 cases of simple voice clearing, 18 cases reached imaging atlantoaxial subluxation, 13 cases had abnormal imaging, and the atlanto-odontoid space on both sides was not equal, but ADI ≤ 5 mm.

Clinically, different examination methods are often adopted according to the degree of cooperation of the children. It is usually based on the degree of deviation of the spinous process of the axis in the cervical spine X-ray of the open-mouth position. However, children are often difficult to cooperate due to forced posture, especially young children, and the success rate of radiography is lower. Accordingly, Wang et al^[10]^ proposed that the examination method could be selected according to the age of children, and X-ray examination was the first choice for children over 7 years old with high cooperation and no nerve damage. For children under 4 years old who were difficult to cooperate, CT plain scan combined with 3-dimensional reconstruction was selected. X-ray or CT examination could be determined according to the cooperation degree of children aged 4 to 7 years. Among them, children with highly suspected cervical spinal cord or ligament injury needed magnetic resonance imaging examination.^[11]^ In this case, due to the serious rotation and dislocation of the child, combined with the bone overlap after rotation, the X-ray could not be diagnosed because of bone overlap, so 3-dimensional CT should be the first choice for children with very serious atlantoaxial dislocation. Therefore, correct shooting, appropriate selection and accurate interpretation of imaging data is one of the important influencing factors for clinical diagnosis of this disease. According to “Tachdjian Pediatric Orthopaedics”^[12]^ and “Spinal Imaging Diagnostics,”^[13]^ the diagnostic criteria of atlantoaxial subluxation in children are preliminarily developed: (1) A recent history of upper respiratory tract infection or other oral infection with cough, sore throat, nasal congestion, runny nose and other manifestations; (2) congential malformations, dysplasia, history of trauma or sudden abnormal activity; (3) clinical presentation typically includes torticollis, neck pain with restricted range of motion, palpable cervical muscle spasm, and tenderness in the mastoid or occipital regions; (4) X-ray of the open-mouth position cervical spine or cervical CT scan combined with 3-dimensional reconstruction show: asymmetrical atlanto-dental interval and significant rotational displacement of the atlantoaxial vertebrae. The diagnosis of atlantoaxial subluxation in children requires the integrity and unity of clinical symptoms, signs and imaging studies. In addition, if the confirmatory treatment is effective, the diagnosis is more accurate.^[14]^

In terms of treatment, there are 3 primary treatments for atlantoaxial subluxation: cervical traction, manual manipulation and surgical intervention. For the former, the children are difficult to cooperate and do not have a targeted to correct the dislocation of the joint, resulting in frequently unsatisfactory therapeutic outcomes; while the latter carries significant risks, substantial trauma, elevated costs, and postoperative limitations in cervical spine mobility, often rendering it less acceptable to patients. In this case, despite the child had been given multiple therapeutic attempts, including cervical collar fixation and traction therapy, the injury failed to resolve. However, manual manipulation, rooted in traditional Chinese medicine principles, offers unique advantages, including meridian regulation, soft tissue adhesion release, joint realignment, muscle spasm relief, and promotion of Qi and blood circulation.^[15]^ The effectiveness of reduction manipulation is important, but safety is more important. At present, the majority of the manual manipulations are pulling method, that is, after rotating a certain angle, adding a certain flash power, increasing the rotation by 3 to 5 degree, which is easy to exceed the physiological limit of the human body and cause new damage, and the pulling method has a high requirement for the doctor’s method, so there are few operators, resulting in discouraging many clinicians. The Nazheng stretch-rotation manipulation used in this case does not need to increase the rotation angle and use suddenly flash power. It is an effective painless bone-setting manipulation for the treatment of simple atlantoaxial subluxation in children. However, it is not suitable for patients with clear surgical indications such as fractures, osseous pathologies and spinal cord compression. This therapeutic approach is effective for acute atlantoaxial subluxation, with successful reduction typically achieved in a single session. For children with long course of disease, soft tissue adhesion and muscular tension imbalance, this therapeutic method should be used after full release of soft tissue, meanwhile, the soft tissue tension should be balanced after reduction to prevent recurrence. There are 2 major changes in this manual manipulation, firstly, the pulling method is changed into the stretching method, significantly enhancing safety parameters; secondly, the state of the doctor’s active treatment and the patient’s passive treatment is transformed into the state of the patient-active participation. The specific operation method and mechanism are as follows (taking the clockwise rotation dislocation of the atlas relative to axis as an example).

**Figure 3.**
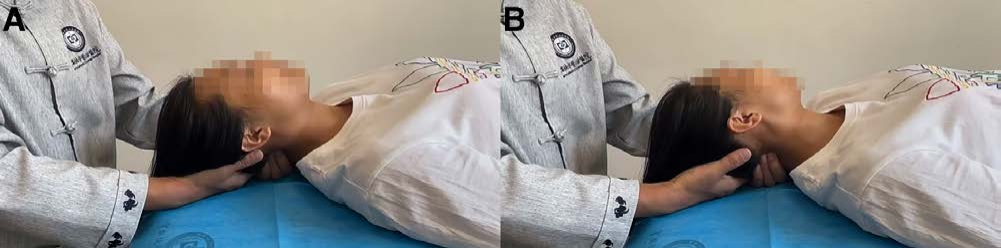
The Nazheng stretch-rotation manipulation operation example.

Firstly, structural abnormalities are assessed to determine the direction and degree of joint dislocation; subsequently, the child is positioned supine, based on the surgeon’s full release of the suboccipital muscle (mainly the rectus capitis posterior major muscle and the obliquus capitis inferior muscle), the surgeon hooked the right side of the spinous process of the axis with one hand and fixed it toward to the left in order to rotate the axis, and the rotational direction is directed to follow the atlas, creating a trend of reduction, the other palm root is close to the occipital bone and gradually stretches the cervical spine upward to give the opportunity of reduction (Fig. 3A). During traction, the compression of the atlantoaxial joint is relatively relieved, then the child is instructed to actively rotate toward the opposite direction of the dislocation to maximal range (Fig. 3B), maintain for 5 seconds, return to neutral (Fig. 3A), and repeat the maneuver. This maneuver can be repeated for 3 to 5 cycles to achieve the purpose of slow reduction of atlantoaxial joint. We create the trends and opportunities for joint reduction, allowing children to achieve reduction with the force of their own head rotation, enhancing children acceptance. This therapy is noninvasive, painless, and has no functional limitation after recovery. It is not only efficient and quick-acting, but also has high safety without pulling method. And it should be the preferred treatment option of this disease, which is worthy of further clinical promotion and research.

## 4. Conclusion

In conclusion, this case mainly used the Nazheng stretch-rotation manipulation to treat the atlantoaxial subluxation in children, which provided a solid clinical basis for the treatment of atlantoaxial subluxation in children, especially providing a new strategy for its treatment. Compared with other treatment methods, the Nazheng stretch-rotation manipulation demonstrates enhanced operational simpleness, superior safety profile, improved therapeutic efficacy, and greater pediatric patient compliance, and it exhibits broad clinical applicability across various practitioner experience levels, including novice clinicians.

## Acknowledgments

The authors would like to thank the patient and his guardians.

## Author contributions

**Conceptualization:** Jiayi Liu, Wei Qi.

**Methodology:** Jiahe Liu, Hongpeng Lin, Qingjun Zhang.

**Resources:** Jiahe Liu.

**Supervision:** Chenxi Qi.

**Writing – original draft:** Jiahe Liu, Jiayi Liu.

**Writing – review & editing:** Yi Zhou.
